# Q&A: Who is *H. sapiens *really, and how do we know?

**DOI:** 10.1186/1741-7007-9-20

**Published:** 2011-03-31

**Authors:** Mason Liang, Rasmus Nielsen

**Affiliations:** 1 Department of Integrative Biology, University of California, Berkeley, California, USA

## Is it true that modern humans have Neanderthals and other archaic species in their direct ancestry?

According to two recently published papers by Green *et al*. and Reich *et al*., the answer to this question is yes. Human genomes are in part composed of DNA from other archaic hominin species that traditionally have not been counted among our ancestors, although the proportion of archaic DNA in the genome depends on your ethnicity. On the basis of analyses of ancient DNA, Green *et al*. report that, on average, 1 to 5% of the genomes of non-African individuals are descended from a Neanderthal, and Reich *et al*. report that 4 to 6% of the genomes of Melanesians are derived from a newly discovered archaic hominin population dubbed the Denisovans. Denisovans and Neanderthals are the only archaic species investigated so far, but future investigations may reveal contributions of DNA from other species, perhaps even from species that have never been characterized well morphologically.

## What is an archaic hominin, exactly?

Hominins are humans and their closely related extinct ancestors. Denisovans and Neanderthals were hominins that last lived approximately 30,000 years ago. Neanderthal fossils were first found in 1856, in the Neander Valley, which lends its name to the species. Since then, specimens have been found in a wide geographical range, including the Middle East, Central Asia, and Western and Central Europe. To date, the only discovered Denisovan remains are the finger bone and two teeth discovered in Denisova Cave in Siberia. On the basis of genetic analysis of the finger bone, Reich *et al*. conclude that Denisovans represent a deeply diverged population distinct from other Neanderthals. Whether Neanderthals and Denisovans comprise separate species is probably mainly an issue of semantics and, in any case, cannot be answered without additional Denisovan samples.

## How does this fit with current theories of human origins?

**Figure 1:**
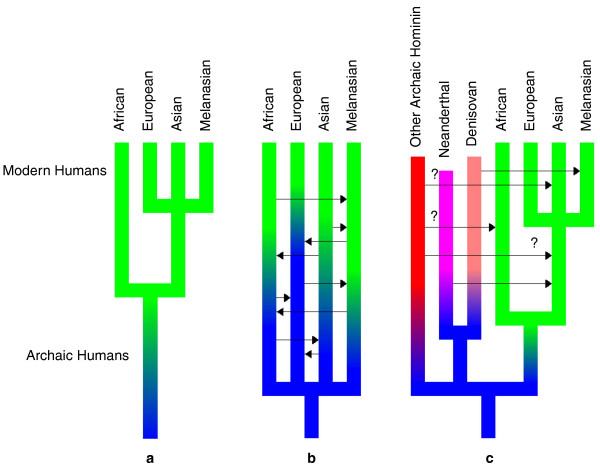
**Human origins**. Each panel shows a hypothesis for the evolutionary history of humans. The colored bars show the phylogenetic relationships between species, with each color representing a species and blue representing the ancestral hominin species. Arrows represent gene flow, or admixture, with question marks to indicate possible admixture from as yet undiscovered hominins. **(a) **The Out of Africa (OOA) hypothesis; **(b) **the multiregional hypothesis; **(c) **a modification of the OOA hypothesis to include the archaic admixture inferred from recent work.

The question of human origins has intrigued scientists ever since Darwin first proposed the theory of evolution. Historically, most of the debate has focused on two competing hypotheses: the out of Africa (OOA) theory (Figure 1a) and the multi-regional theory (Figure 1b). The OOA hypothesis posits that anatomically modern humans first evolved in Africa 200,000 to 150,000 years ago and then migrated out of Africa 100,000 to 60,000 years ago, displacing other archaic hominins, and giving rise to all current human populations. The multi-regional theory suggests that archaic hominins spread out of Africa much earlier, and that humans then evolved from this Eurasia-wide population, with some degree of interbreeding, and thus gene flow, among individuals from different populations being responsible for the degree of genetic differentiation between populations we currently observe. Mitochondrial (mt) DNA data first reported in 1987 and subsequent analyses of autosomal DNA seemed to support the OOA hypothesis.

However, even before the publication of the Neanderthal genome, analyses of modern human DNA from different geographic sources by Jeffrey Wall and others had suggested that, contrary to the earlier consensus, anatomically modern humans evolved in Africa recently, but admixed with endemic archaic hominids - Neanderthals, Denisovans, or even *Homo erectus *- as they spread throughout the world (Figure 1c), and that ancestral admixture may be much more common than previously thought.

Wall *et al*. based their analysis on the pattern of haplotype lengths. After controlling for other confounding factors, such as demographic history and recombination rate variation, they concluded that the observed lengths of these regions could only be accounted for by archaic admixture on the order of 5%. The evidence of admixture from Neanderthal and Denisovan nuclear DNA lends credence to these claims.

## Isn't it extremely difficult to get authentic undamaged DNA from individuals dead for over 30,000 years?

Yes. It was necessary to locate samples that had been buried in cool and dry conditions, under which DNA is degraded relatively slowly. Even so, for the Neanderthal samples, most DNA fragments were very short, and approximately 95 to 99% of the DNA in the samples belonged to bacteria. To reduce the amount of sequencing needed, the relative proportion of hominin DNA was increased by treating the DNA extract with a concoction of restriction enzymes that were chosen to cut bacterial DNA preferentially. This increased the relative proportion of Neanderthal DNA to over 10%. This enriched extract was analyzed using new-generation sequencing machines, which produced a draft sequence with approximately 1.3× coverage - that is, on average, each base pair in the genome was sequenced 1.3 times. Because of the random nature of next-generation sequencing, this means that certain parts of the genome will not have been sequenced at all, while other parts will have been sequenced many more times.

The genetic material from the Denisovan individual was extracted from a finger bone. Because of the cooler climate in Siberia, there was less environmental degradation of the DNA. However, the small volume of the finger bone yielded only enough DNA to sequence the Denisovan genome to 1.9× coverage.

## We must have a lot of DNA in common with archaic hominins because of our shared ancestry - How can we infer interbreeding?

If we have two human populations, one of which has undergone more archaic admixture than the other, then we expect the more admixed human population to be more genetically similar to the archaic hominin than the other human population. This intuition is formalized by the ABBA-BABA test. In this statistical test, DNA representing the same sites in a chimpanzee sequence, an archaic hominin sequence, and sequences from a pair of modern human populations, such as Han and Yoruban or Japanese and French, designated H1 and H2, are compared. Only sites with two alleles, A and B, are considered. The chimpanzee is assumed to carry A, the ancestral allele. Two numbers are then computed, n_ABBA_, the number of sites where the chimpanzee and one of the pair of modern humans (H2) have allele A and the archaic hominin and the other modern human (H1) have allele B (ABBA) and n_BABA_, the number of sites where chimpanzee and H1 have allele A and the archaic hominin and H2 have allele B (BABA). Finally, n_ABBA _and n_BABA _are added up over all pairings of H1/H2 samples from the two human populations being analyzed. If there had been no archaic admixture, then the difference of these sums is expected to be 0. If the difference is significantly different from 0, then the null hypothesis of no admixture is rejected. Using population genetic models, the admixture fraction can also be estimated from the magnitude of this difference. The ABBA-BABA test can then be used for each pair of human populations to determine the differences in admixture rates between them.

The results of the ABBA-BABA test showed that human non-African populations are more closely related to Neanderthals than African populations. When applied to the Denisovan genome, Reich *et al*. found that only Melanesians showed evidence of admixture.

## This seems quite a subtle test - Might these results be explained by human contamination?

Probably not. Contamination is a serious problem in any sequencing project. A recent paper by Longo *et al*. reports significant human contamination in non-primate genome databases, and previous analyses of Neanderthal genetic material have also been plagued by human DNA contamination.

In the light of this earlier experience, researchers took several precautions to guard against contamination. The initial sample preparation and DNA extraction were done in a clean room, using several procedures to reduce the chances of modern human DNA contamination. As an additional step in the sample preparation, special primers were ligated onto both ends of each fragment, identifying the fragments. During the sequencing, only reads with this clean room tag were used to assemble the draft genome, minimizing the effect of post-clean room contamination.

The efficacy of these methods was validated using three different procedures: by looking at mtDNA; by looking for Y chromosome sequences; and by using statistical analyses of autosomes. mtDNA is much easier to sequence because it occurs in much higher concentration than nuclear DNA. As a result, the Neanderthal mtDNA sequence can be very accurately determined, and several fixed differences between humans and Neanderthals have been identified. These differences can be used to estimate the ratio of human mtDNA to Neanderthal mtDNA in the sample. Likewise, because all the samples were female, the amount of Y chromosomal DNA can be used to estimate the level of contamination from human males. Finally, researchers used human heterozygosity and allele frequency data to directly estimate contamination in the autosomal DNA. All three methods estimated the human contamination to be 1% or less.

This is consistent with the results of a blind test in which Green *et al*. examined present-day human genetic variation without knowledge of the Neanderthal sequence, and were able to locate regions of the human genome that appeared admixed. Comparison of their predictions with the Neanderthal data showed that these candidate regions matched the Neanderthal sequence at a higher frequency than could be explained by any level of contamination.

## What about DNA damage?

The main problem in dealing with ancient DNA is the dearth of genetic material. The Neanderthal and Denisovan genomes could not be sequenced to a higher coverage not because of a lack of money or time, but because of a lack of DNA extract; the three bones from Vindija Cave and the one from Denisova Cave have been completely hollowed out to produce the genomes reported.

Ancient DNA sequencing typically shows a much high error rate than observed in modern DNA. Errors in the reported genome can be caused by degradation of the DNA from the environment or by sequencing error. In ancient DNA samples, deamination of cytosine residues causes C to have the chemical properties of T, and G to have the chemical properties of A. As a result, the Neanderthal draft genome shows an abnormally large number of C-> T and G-> A substitutions, the vast majority of which are errors. In sequencing the Denisovan samples, this deamination was chemically reversed, allowing the C and G residues to be sequenced correctly. This, together with the drier and cooler climate at Denisova Cave, resulted in DNA samples that were about ten times less damaged.

Sequencing error can also be a problem, as the error rate of new-generation sequencing is only slightly lower than the divergence between humans and Neanderthals. However, this problem will hopefully disappear as new-generation sequencing technology becomes more accurate and the discovery of new samples allows for deeper coverage.

## That sounds serious - How confident can we be of any interpretation if the sequencing error rate and the divergence are that close?

The statistical analysis of the Neanderthal and Denisovan genomes was designed with the limitations of the data in mind. A paper by Durand *et al*. argues that the ABBA-BABBA test for admixture is not sensitive to confounding factors, such as human or Neanderthal demographic history, sequencing error or damage to the DNA, as long as the H1 and H2 samples were processed in the same way. However, one source of concern is the possibility of a shared error structure caused by DNA sequencing methods. Current sequencing technology is highly temperamental, and the frequency and type of sequencing errors in the final data depend on many factors, such as sample preparation, the type of sequencing machine, contamination from local conditions and reagents, and sequencing coverage. If the error structures of the archaic DNA and one of the modern human DNA samples are similar to each other for one of many reasons, the ABBA-BABA test could report admixture when it did not in fact occur. Even a very small proportion of shared errors could cause a strong effect on the ABBA-BABA statistic. For example, small effects that we typically tend to ignore, such as shared contamination of reagents between the samples, could cause artifactual evidence of admixture. Green *et al*. and Reich *et al*. made great efforts to control for these effects, and appear to have succeeded. However, the issues of errors in next-generation sequencing data, particularly for ancient DNA, and their consequences for current and future inference of low levels of admixture remain a critical issue that is likely to be the focus of much future research.

## Assuming that we can be confident of the conclusions of these studies, how much of our genomes comes from other hominins?

These two papers only investigated the possibility of admixture from Neanderthals and Denisovans into humans. It is possible that other archaic hominins, perhaps as yet undiscovered, also contributed to the human genome. In fact, Plagnol and Wall report that there is evidence for significant admixture into African populations as well, although no candidate species has been proposed.

On the basis of the data and analyses presented by Green *et al*. and Reich *et al*., it appears that a simple out of Africa hypothesis with no admixture does not give the full picture of human origins. As sequencing technology improves and additional archaeological discoveries are made, we should be able to gain a more detailed understanding of what now seems to be the mosaic ancestry of the human genome.

## Where can I find out more?

1. Green RE, Krause J, Briggs AW, Maricic T, Stenzel U, Kircher M, Patterson N, Li H, Zhai W, Fritz MH, Hansen NF, Durand EY, Malaspinas AS, Jensen JD, Marques-Bonet T, Alkan C, Prüfer K, Meyer M, Burbano HA, Good JM, Schultz R, Aximu-Petri A, Butthof A, Höber B, Höffner B, Siegemund M, Weihmann A, Nusbaum C, Lander ES, Russ C, *et al*.: **A draft sequence of the Neandertal genome**. *Science *2010, **328:**710-722.

2. Reich D, Green RE, Kircher M, Krause J, Patterson N, Durand EY, Viola B, Briggs AW, Stenzel U, Johnson PL, Maricic T, Good JM, Marques-Bonet T, Alkan C, Fu Q, Mallick S, Li H, Meyer M, Eichler EE, Stoneking M, Richards M, Talamo S, Shunkov MV, Derevianko AP, Hublin JJ, Kelso J, Slatkin M, Pääbo S: **Genetic history of an archaic hominin group from Denisova Cave in Siberia**. *Nature *2010, **468:**1053-1060.

3. Durand E, Patterson N, Reich D, Slatkin M: **Testing for ancient admixture between closely related species**. *Genetics *2011, in press.

4. Wall J, Hammer M: **Archaic admixture in the human genome**. *Curr Opinin Genet Dev *2006, **16:**606-610.

5. Plagnol V, Wall J: Possible ancestral structure in human populations. *PLoS Genet *2006, 2:972-979.

